# Transcriptional analysis of viral mRNAs reveals common transcription patterns in cells infected by five different filoviruses

**DOI:** 10.1371/journal.pone.0201827

**Published:** 2018-08-02

**Authors:** César G. Albariño, Lisa Wiggleton Guerrero, Ayan K. Chakrabarti, Stuart T. Nichol

**Affiliations:** Viral Special Pathogens Branch, Centers for Disease Control and Prevention, Atlanta, GA, United States of America; University of Texas Medical Branch at Galveston, UNITED STATES

## Abstract

Filoviruses are notorious viral pathogens responsible for high-consequence diseases in humans and non-human primates. Transcription of filovirus mRNA shares several common features with transcription in other non-segmented negative-strand viruses, including differential expression of genes located across the viral genome. Transcriptional patterns of Ebola virus (EBOV) and Marburg virus (MARV) have been previously described using traditional, laborious methods, such as northern blots and in vivo labeling of viral mRNAs. More recently, however, the availability of the next generation sequencing (NGS) technology has offered a more straightforward approach to assess transcriptional patterns. In this report, we analyzed the transcription patterns of four ebolaviruses—EBOV, Sudan (SUDV), Bundibugyo (BDBV), and Reston (RESTV) viruses—in two different cell lines using standard NGS library preparation and sequencing protocols. In agreement with previous reports mainly focused on EBOV and MARV, the remaining filoviruses used in this study also showed a consistent transcription pattern, with only minor variations between the different viruses. We have also analyzed the proportions of the three mRNAs transcribed from the GP gene, which are characteristic of the genus *Ebolavirus* and encode the glycoprotein (GP), the soluble GP (sGP), and the small soluble GP (ssGP). In addition, we used NGS methodology to analyze the transcription pattern of two previously described recombinant MARV. This analysis allowed us to correct our construction design, and to make an improved version of the original MARV expressing reporter genes.

## Introduction

The family *Filoviridae* includes several viruses, such as Ebola (EBOV), Sudan (SUDV), Bundibugyo (BDBV), Marburg (MARV), Ravn (RAVV), and Taï Forest viruses, that are responsible for sporadic outbreaks of hemorrhagic fever on the African continent, and often result in fatal disease in humans and non-human primates. Two others filoviruses, Reston virus (RESTV) and Lloviu virus (LLOV), have not been associated with human disease to date [1–3].

Filoviruses carry a single-stranded, negative-sense RNA genome that is approximately 19 kb long and encodes seven genes: nucleoprotein (NP), viral protein 35 (VP35), VP40, glycoprotein (GP), VP30, VP24, and RNA-dependent RNA polymerase (L), all separated by intergenic regions (IGR) [1]. During replication inside infected cells, transcription of filoviral genes occurs via a mechanism similar to that of other non-segmented negative-strand (NNS) viruses [1, 4]. The viral genes are transcribed sequentially from the 3′ end to the 5′ end of the viral genome, with the viral polymerase stopping and restarting at each IGR. Transcription stop and re-start signals in the IGR of the filoviral genome are very highly conserved and are similar to those in other NNS viruses. As in other NNS viruses, transcription of the filovirus genome gradually decreases each time the viral polymerase proceeds to the next downstream gene, thus generating a gradient of mRNA abundance (NP > VP35 > VP40 > GP > VP30 > VP24 > L) inside infected cells [1, 5].

The pattern of filovirus transcription has been traditionally studied using various labor-intensive methods, such as northern blotting, in vivo labeling of viral mRNAs, and cloning and sequencing PCR amplicons [6, 7]. In a more recent report, Shabman and collaborators [8] used a next-generation sequencing (NGS) approach to analyze the transcription of EBOV (Mayinga isolate, Yambuku variant) and MARV (Angola strain) genes. In order to expand these studies to additional filoviruses, we used a similar NGS approach to characterize the viral mRNAs generated in cultured cells infected with EBOV, SUDV, BDBV, or RESTV. Moreover, in these ebolaviruses, we analyzed the sequence variants generated by editing GP mRNAs. Finally, we used the NGS approach to confirm the abnormal transcription pattern of a recombinant MARV expressing a green fluorescent protein that was previously generated in our lab. With the newly acquired information, we were able to change the construct design of these reporter MARV and to make an improved version of the original virus that exhibits a transcriptional pattern more similar to that of the wild-type virus.

## Materials and methods

### Cell culture and biosafety

All work with infectious viruses was performed in a biosafety level 4 (BSL-4) facility. Huh7 (human liver cells, obtained from Apath, LLC) and Vero-E6 (African green monkey kidney cells, obtained from ATCC) cells were propagated in Dulbecco’s modified Eagle’s medium (DMEM) supplemented with 5% fetal bovine serum, penicillin-streptomycin, and non-essential amino acids (Huh7 only).

### Human macrophages

Peripheral blood mononuclear cells were obtained from apheresis products of healthy human donors by Ficoll layering, centrifugation, and extraction of the buffy coat. Monocytes were selected through depletion using the human Monocyte Isolation Kit II (Miltenyi Biotec). Monocytes were cultured in RPMI media with 10% fetal bovine serum and penicillin/streptomycin for 7–10 days to allow maturation into macrophages.

### Ethics statement

The use of human blood products was approved by Emory University Institutional Review Board (IRB reference IRB00045947). Under this protocol, no donor personal information was provided, and informed consent was given. All adult blood donors provided informed consent, and a parent or guardian of any child participant provided informed consent on the child’s behalf.

### Viruses

Four wild-type ebolaviruses were used in this study: EBOV, SUDV, BDBV, and RESTV. Huh7 and human macrophages seeded in 12-well plates were infected with the indicated viruses at multiplicity of infection (moi) = 0.1. Singleton infections were done for each virus in both cell types. Viruses were adsorbed for 1 h, complete media was added, and virus propagation was allowed for 1–3 days post infection (dpi) at 37°C. Supernatants and cell monolayers were harvested, and total RNA or purified mRNA was used as templates for the cDNA libraries.

### Effect of high temperature

Vero-E6 cells seeded in 12-well plates were infected with EBOV, SUDV, BDBV, or RESTV at moi ≥ 2. After 1 h of virus adsorption at 37°C, infected cell cultures were transferred to 37°C or 40°C incubators. Total cellular RNA was harvested 1 dpi, and purified mRNA was used as templates for the cDNA libraries.

### NGS and data analysis

Total RNA from infected cells or from cell supernatants was harvested and extracted with 500 μL of 1× RNA Lysis/Binding Solution Concentrate, followed by magnetic bead purification using the MagMAX Total RNA Isolation kit according to the manufacturer's recommendations (Thermo Fisher). From the total RNA, mRNAs were captured using magnetic oligo-dT beads, and subsequently used to make cDNA libraries using the KAPA mRNA HyperPrep Kit (Roche), then sequenced using paired-end 2 × 150 chemistry on the Illumina Miniseq with a high-output sequencing cartridge. All sequencing details, including the number of mapped reads and the average coverage, are shown in S1 and S2 Tables. Analyses of NGS data, including read mapping and variant detection, were done using CLC Genomics Workbench 9.1 (CLC-GWB, https://www.qiagenbioinformatics.com/products/clc-genomics-workbench/). Variant detection analysis was performed using a maximum cut-off of 1%, so that the less abundant variants were excluded from our analysis. Two relevant controls for the NGS library preparation and variant detection are included in S3 Table: 1) an NGS library made from plasmid DNA containing a full-length cDNA copy of EBOV genome, and 2) an NGS library made from an in vitro transcript generated from the full-length EBOV plasmid.

### Sequence data

Viral sequences are available from GenBank; the accession numbers are KT589389, NC_006432, NC_014373, and KY798007. The raw and processed NGS data are accessible at NCBI's Gene Expression Omnibus (https://www.ncbi.nlm.nih.gov/geo/) and through GEO Series accession number GSE114905.

### Rescue of infectious viruses

Rescue of recombinant MARV was performed in BHK21 cells as described previously [9]. Supernatants from the transfected cells were harvested 4 days post-transfection, clarified by low-speed centrifugation, and passaged twice in Vero-E6 cells. The rescued viruses were sequenced to completion and no unexpected changes were noted.

### qRT-PCR

Matured human macrophages were infected with the indicated viruses, and gene expression was measured using a standard qRT-PCR array (PAHS-122Z, Qiagen) following previously described protocols. Data from representative interferon-related genes were plotted as fold induction over mock-infected cells.

### Growth kinetics

To characterize the growth kinetics of recombinant MARV, human macrophages were infected at moi of 0.02 at 37°C. After 1 h adsorption, monolayers were washed 3 times with PBS to eliminate any residual virus. Aliquots of the supernatant were taken daily, and viral titers were obtained by TCID_50_.

## Results and discussion

**Fig 1:**
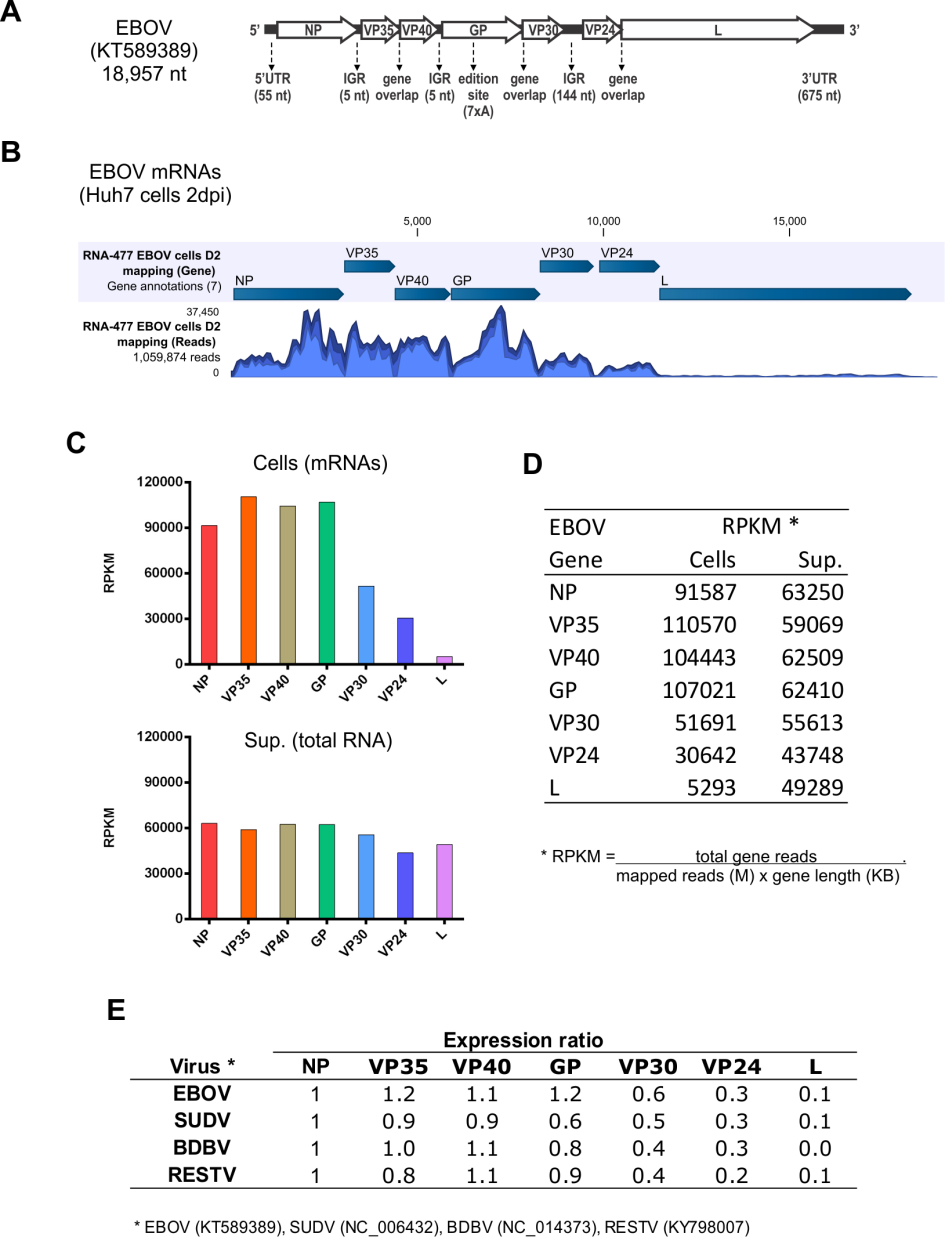
(A) Schematic representation of the genome of EBOV Makona variant (GenBank #KT589389), depicted in the viral complementary sense, with the seven genes shown in the 5′ to 3′ orientation. The lengths of the 5′ and 3′ untranslated regions (UTR), the intergenic regions (IGR), and the glycoprotein (GP) editing site are also indicated. (B) Graphical depiction of the mapped reads corresponding to EBOV mRNAs. Huh7 cells were infected with EBOV at moi = 0.1. Total RNA was harvested 2 days post infection, and purified mRNAs were used to make NGS libraries. (C and D) Graphical depiction and table showing the RPKM analysis of EBOV gene levels. The RPKM index standardizes the number of mapped reads to each gene, normalizing for gene length and for the total mapped reads of the library. “Cells” indicates mRNA isolated from cell lysates; “Sup.” refers to total RNA harvested from supernatants of infected cells. (E) Expression ratio of each viral gene relative to the nucleoprotein (NP) mRNA levels in Huh7 cells infected with EBOV, SUDV, BDBV, or RESTV.

Since human liver cells are an important target of filovirus infection, we first analyzed the viral mRNAs generated during viral propagation in a monolayer of Huh7 cells, a fully differentiated human liver cell line, infected with EBOV (Makona variant; Fig 1A). Briefly, we used mRNAs purified from infected cells or total RNA extracted from cell supernatants to make NGS libraries using standard protocols. All sequencing details, including the number of mapped reads and the average coverage, are shown in S1 Table.

As expected, the graphical depiction of the mapped reads (Fig 1B) showed a differential expression of viral mRNAs in the infected cells. In order to quantify the gene expression, we used RPKM [10], an index that standardizes the number of reads mapped to each gene, by normalizing for the gene length and for the total mapped reads for the sample library. This index facilitates the comparison of mRNA levels from each virus and between different samples. Interestingly, the RPKM analysis (Fig 1C and 1D) showed similarly high expression levels for NP, VP35, VP40, and GP mRNAs, intermediate levels for VP30 and VP24, and lower levels for L. Total RNA extracted from infected cell supernatants was also included as a control to show the even distribution of reads that mapped to the entire EBOV genome.

The expression ratio of EBOV genes (mRNA levels of other genes relative to NP mRNA level) was also compared with that of three other ebolaviruses. As shown in Fig 1E, the four upstream genes (NP to GP) were expressed at similarly high levels, while the three downstream genes (VP30 to L) were expressed in lower levels in Huh7 cells infected with EBOV, BDBV, or RESTV.

**Fig 2:**
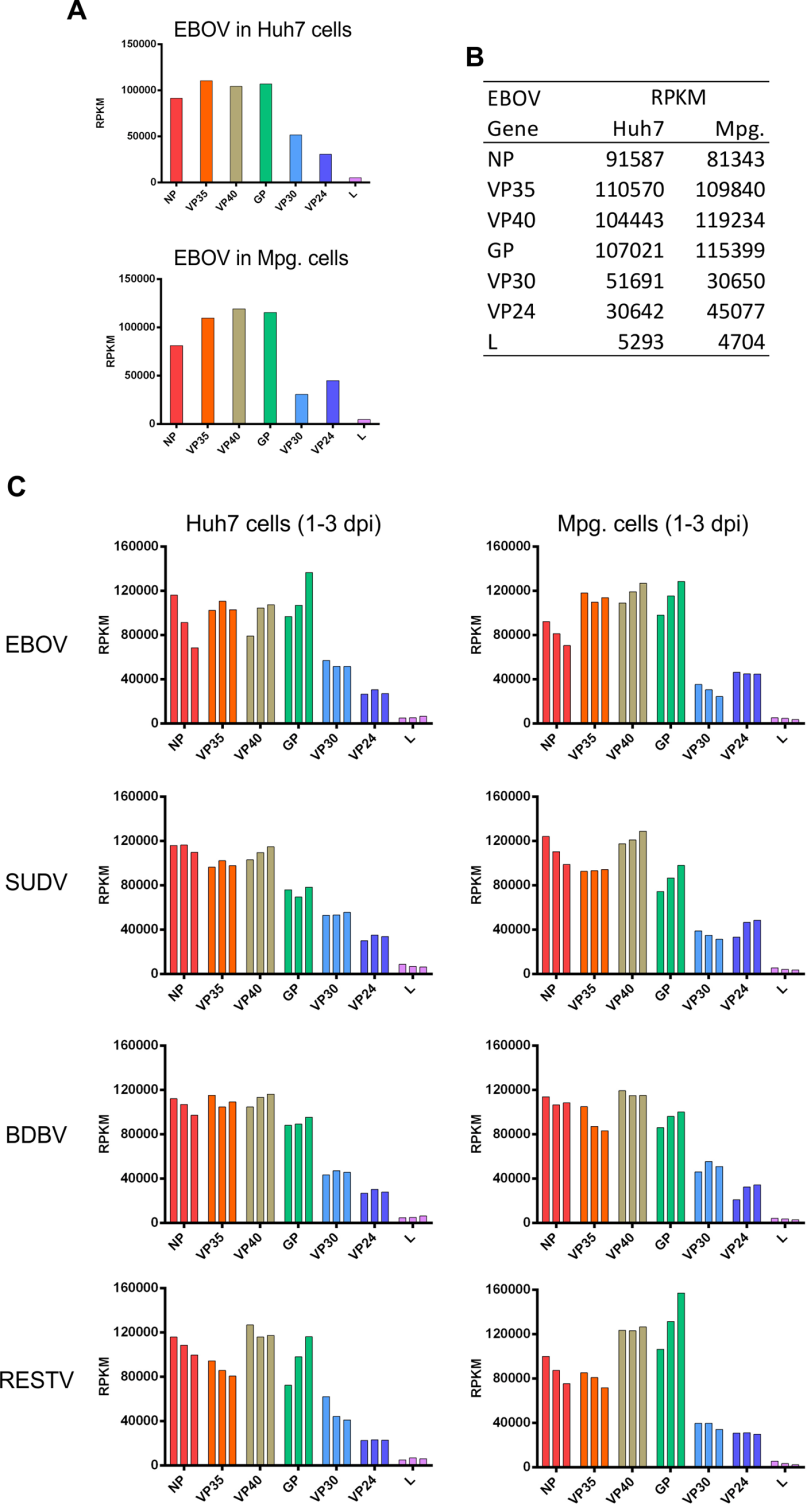
(A and B) Graphical depiction and table showing the RPKM analysis of EBOV gene levels in infected Huh7 and human macrophage (Mpg) cells. (C) Expression of viral genes during propagation of EBOV, SUDV, BDBV, or RESTV in Huh7 or Mpg cells. Both cell types were infected at moi = 0.1, and harvested at 1, 2, or 3 dpi. For each virus, 3 columns are shown, representing each harvesting day. NGS libraries were designed as in Fig 1.

In order to determine whether viral gene expression is affected by different cellular environments, we further compared the transcription pattern in Huh7 cells and primary human macrophages (Mpg cells), which are another primary site of filovirus replication in humans and non-human primates, infected with EBOV, SUDV, BDBV, and RESTV [1]. As shown in Fig 2A and 2B, the pattern of gene expression was mostly similar in Huh7 and Mpg cells infected with EBOV, with only a slight increase in VP24 over VP30 levels in Mpg cells.

We also sought to examine whether the expression of EBOV, SUDV, BDBV, and RESTV genes changes over time in the two cell types. Transcriptional analysis of these viruses in Huh7 and Mpg cells infected at low moi and harvested at 1, 2, or 3 dpi revealed several distinctive features for each of the four ebolaviruses. Viral propagation during the course of the infection can be noted by the increased levels of reads mapping to the viral mRNAs (S1 Table) and to the viral genome (S2 Table). A noticeable change over the course of EBOV and RESTV infection was the simultaneous increase of GP and decrease of NP mRNA levels in both cell types over the first 3 days of infection (Fig 2C). Minor changes in GP and NP mRNA levels were also observed during BDBV and SUDV infection, but their significance was not statistically established. In general, changes in mRNA levels could be due to changes in gene expression, or due to particular stability of an mRNA in the intracellular environment. Independent of the underlying mechanism, it is reasonable to speculate that increased levels of GP mRNA from 1 to 3 dpi could be advantageous to the virus in later stages of the viral cycle.

**Fig 3:**
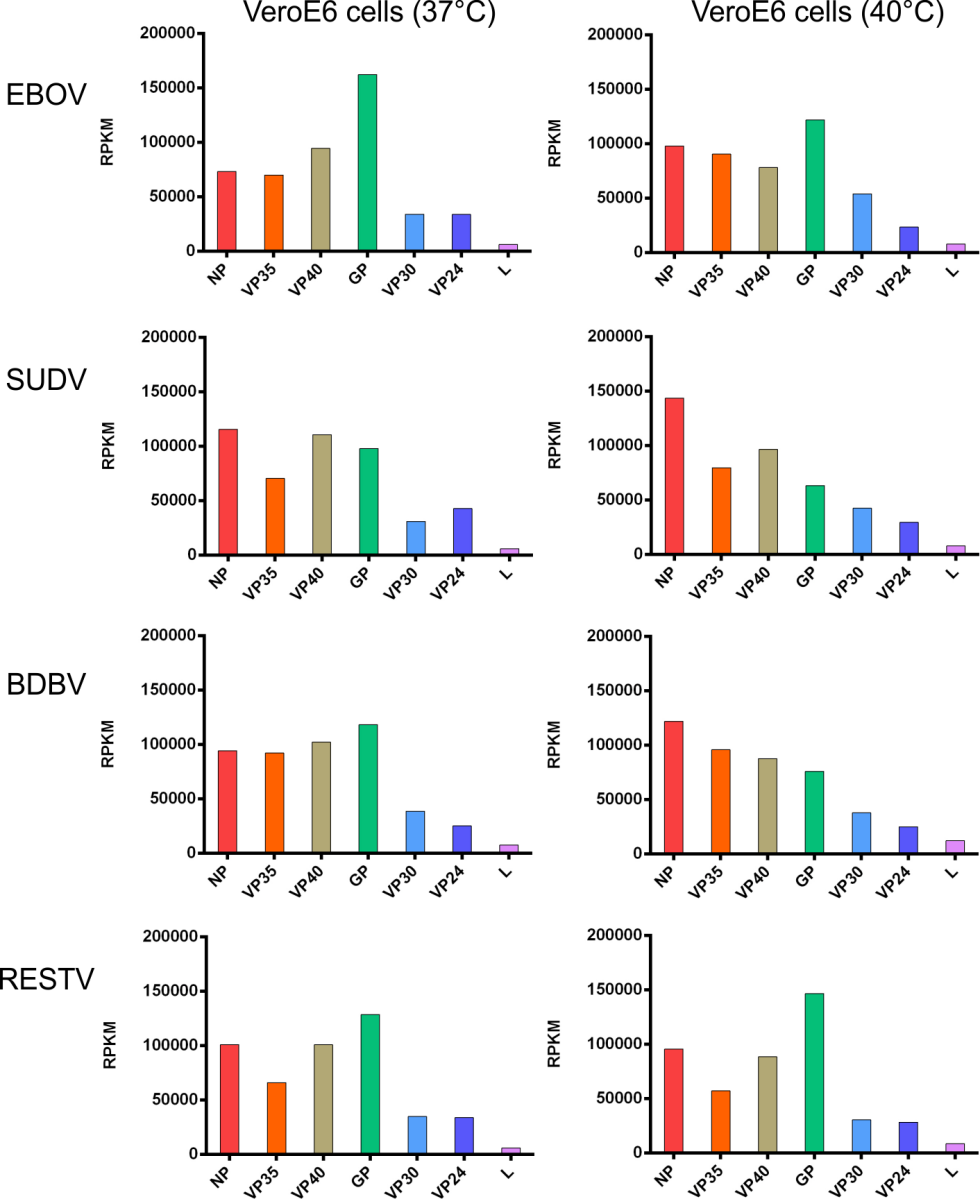
Effects of incubation temperature on viral mRNA levels. Vero-E6 cells were infected with EBOV, SUDV, BDBV, or RESTV at moi ≥ 2. After 1 h of virus adsorption at 37°C, infected cell cultures were incubated at 37°C or 40°C. Infected cells were harvested at 1 dpi. NGS libraries and RPKM analysis were done as in Fig 1.

We further investigated whether mRNA levels would also change when viruses replicated at an abnormally high temperature, like in a febrile patient. For this test, Vero-E6 cells were infected with EBOV, SUDV, BDBV, or RESTV for 1 h at 37°C to allow virus adsorption, and then incubated at 37°C or 40°C for 24 h. RPKM analysis (Fig 3) indicated that the patterns of gene expression were similar at both temperatures for EBOV, SUDV, and RESTV. In contrast, transcription pattern of BDBV differed with temperature. The smooth gradient of gene expression observed at 40°C suggested that the high temperature may affect the efficiency with which the BDBV viral polymerase complex recognizes the start-stop signals present in the intergenic regions.

**Fig 4:**
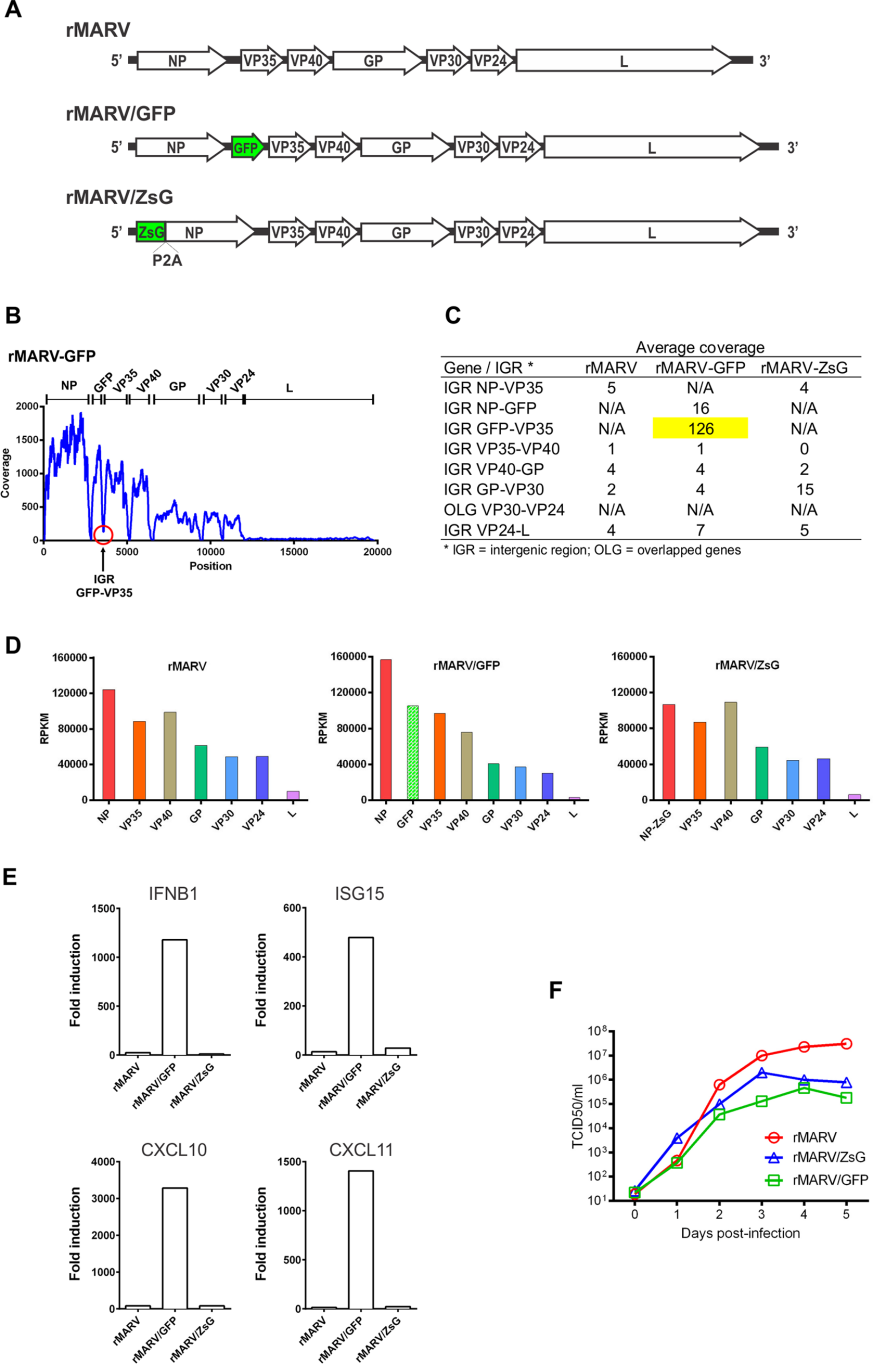
(A) Schematic representation of recombinant MARV genomes, depicted in the viral complementary sense. (B) Coverage analysis across the genome of rMARV-GFP. The area of unusually high coverage in the second IGR is indicated with an arrow. (C) Average coverage in each intergenic region of the three recombinant MARV viruses. (D) MARV mRNA levels in human macrophage (Mpg) cells infected with wild-type recombinant MARV (rMARV), rMARV expressing GFP (rMARV/GFP, previously created), or rMARV expressing ZsGreen (rMARV/ZsG, designed in this study). Cells were infected with the indicated viruses at moi = 1. Total RNA was harvested 1 dpi and NGS libraries were created as in Fig 1. (E) Cellular antiviral response. Human macrophages were infected with the indicated viruses, and gene expression was measured using a standard qRT-PCR array. Data from representative interferon-related genes were plotted as fold induction over mock-infected cells. (F) Growth kinetics of recombinant MARV. Human macrophages were infected with rMARV, rMARV/GFP, or rMARV-ZsG, and viral titers were determined by TCID_50_ assay in Vero-E6 cells.

Moreover, we also used the NGS approach to examine the transcription profiles of three recombinant MARV that were made in our lab (Fig 4A). In particular, the previously described rMARV/GFP exhibited reduced growth when compared to the wild-type virus (rMARV) in infected human Mpg cells [9]. This phenotype was most likely due to the insertion of an additional transcription unit (ATU) to express GFP into the NP-VP35 intergenic region, and reduced expression of downstream genes.

Interestingly, the coverage analysis of rMARV-GFP (Fig 4B) revealed unusually high coverage in the second IGR between the GFP and VP35 genes. The reads mapped to this IGR may correspond to a read-through transcript that failed to terminate at the stop signal. As reported for filoviruses and other NNS RNA viruses [4, 11–13], this bicistronic mRNA would exhibit high expression of the upstream ORF (GFP) due to the proximity of the capped 5′ mRNA end, and lower expression of the downstream ORF (VP35). The average coverage in each IGR of the three recombinant MARV viruses is also shown in Fig 4C. The unusually high coverage on an IGR was not found anywhere else in this virus or in the wild-type virus.

We hypothesized that the insertion of an additional transcription unit (ATU) perturbed the natural order of transcription, and possibly decreased the levels of the viral proteins with an innate immune-modulatory functions, such as VP35 and VP40 [14, 15]. This hypothesis is also consistent with a previous report describing the low pathogenicity of GFP-containing EBOV in infected mice and NHPs [16]. In order to correct the abnormal phenotype of the recombinant MARV expressing a reporter gene, we made a new recombinant MARV expressing ZsGreen (rMARV/ZsG) using a genomic design similar to other viruses recently made in our lab [17–20]. In this design, the reporter gene is fused to the self-cleaving peptide P2A and to NP, and the encoded proteins are later released through the action of P2A (Fig 4A). In contrast to rMARV/GFP, the new rMARV/ZsG exhibited a transcription profile (Fig 4D) similar to that of the wild-type rMARV, and did not elicit increased expression of antiviral genes in infected Mpg cells (Fig 4E). Characterization of the growth kinetics of the three recombinant viruses (Fig 4F) showed that rMARV/ZsG grew to higher levels than rMARV/GFP, but still lower than wild-type rMARV. An incomplete release of NP from the original fusion protein (ZsG-P2A-NP) may explain the slightly reduced growth of rMARV/ZsG in comparison to rMARV, an inherent characteristic that was previously observed in similar viral constructs [17, 19].

Besides the seven major mRNAs encoded in the filovirus genome, EBOV has an additional transcriptional feature: editing GP mRNA generates alternative protein products. GP mRNA editing is thought to be common to other members of the genus *Ebolavirus* (SUDV, BDBV, RESTV, TAFV), but not to those of the genus *Marburgvirus* (MARV and RAVV) [1, 5]. GP mRNA editing takes place by the addition of non-templated adenosines (A) on a stretch of 7×A (7×U in the vRNA template). As a result, two major products are generated: the most abundant mRNA, which is a perfect copy of the vRNA template (7×A mRNA), encoding a soluble form of the glycoprotein (sGP); and a less abundant edited mRNA with 8×A at the editing site, which codes for the mature glycoprotein (GP). An additional minor mRNA species carries 9×A at the editing site, and encodes a short form of the soluble glycoprotein (ssGP) [21]. The mechanism of mRNA editing and the requirements of cis-acting signals has been previously studied by others [21, 22].

In order to examine the GP-derived mRNAs from EBOV, SUDV, BDBV, and RESTV, we compared the frequency of the major GP variants containing insertions or deletions (InDel variants) present in infected Huh7 and Mpg cells (Table 1). For EBOV, the ratios of the most common GP variants containing 7, 8, and 9 A residues at the canonical editing site were slightly different in Huh7 (~67:20:2 of 7×A:8×A:9×A) than in Mpg (~61:19:2) cells. Minor differences in EBOV GP mRNA ratios were also found by Shabman and collaborators [8] in Vero-E6 (~72:26:1) and Thp1 (~68:28:2) cells. Interestingly, the relative abundance of ssGP mRNA estimated by NGS analysis (~2% in our report, and 1–2% in Shabman’s report) is somewhat lower than that the values (4–5%) originally determined using a traditional RT-PCR and Sanger sequencing approach [21].

**Table 1:** InDel variants at the canonical GP editing site. Huh7 and Mpg cells were infected with EBOV, SUDV, BDBV, or RESTV at moi = 0.1. Total RNA was harvested 3 dpi, and purified mRNAs were used to make NGS libraries. Variant detection was done using a minimum cut-off of 1%.

InDel variants at GP editing site					
Reference			Predicted	Frequency (%)	
sequence	Position	Allele	protein	Huh7	Mpg
EBOV	6918	7A	sGP	66.8	61.4
		8A	GP	19.5	19.1
		9A	ssGP	1.5	2.3
SUDV	6877	7A	sGP	56.1	57.1
		8A	GP	17.2	20.6
		9A	ssGP	2.3	2.6
		10A	sGP+1aa	2.8	1.9
		11A	GP+1aa	1.2	−
		12A	ssGP+1aa	−	1.1
BDBV	6900	7A	sGP	69.5	62.1
		8A	GP	19.2	23.3
		9A	ssGP	1.4	2.3
		10A	sGP+1aa	−	−
		11A	GP+1aa	−	1.1
RESTV	6912	7A	sGP	78.1	71.8
		8A	GP	14.4	16.8
		9A	ssGP	3.0	3.0
		10A	sGP+1aa	−	1.4

In addition to the GP mRNAs containing 7–9 A at the GP editing site, other minor species containing 10–12 A residues were also detected in cells infected with SUDV, BDBV, and RESTV (Table 1). These minor mRNA species are expected to be translated to proteins similar to sGP, GP, and ssGP, but containing 1 additional amino acid (aa) residue. Thus, mRNAs containing 10, 11, or 12 A residues would code for sGP + 1 aa, GP +1 aa, and ssGP + 1 aa, respectively.

In agreement with the report by Shabman and collaborators [8], our NGS analysis also revealed additional mRNA variants generated outside the canonical editing site of EBOV. These minor variants appeared to be generated at 6–7 homopolymer stretches present throughout the entire genome of EBOV (S3 Table). Overall, these edited mRNAs could potentially generate truncated forms of the wild-type proteins, but the relevance of these products is beyond the scope of our investigation.

### Conclusions

Using standard NGS protocols allowed us to quantitate viral transcripts in two different cell types infected with four wild-type ebolaviruses and 3 recombinant marburgviruses. Although the filoviruses used in our investigation were expected to display a similar transcription pattern, the confirmation of these results using a traditional, non-NGS approach would have been extremely laborious and would have required a complicated standardization process. For example, it would have been necessary to standardize seven riboprobes (1 per gene) to be used on northern blots, or seven qPCR assays, for each virus used in the study. Moreover, the use of the current NGS technology also allowed us to quantify the major mRNA variants transcribed from the GP gene of the four ebolaviruses. Finally, we used the same technical approach to revisit the abnormal phenotype of a recombinant MARV previously made in our lab, and to make a new, improved version of a recombinant MARV expressing a reporter gene. Similar to an equivalent version of a recombinant EBOV expressing ZsG that we previously described [17], the new rMARV/ZsG will be used in future studies to screen antiviral compounds and to test the presence of neutralizing antibodies in samples from infected bats or other wild or laboratory animals.

## Supporting information

S1 Table NGS mapping details corresponding to each infected cell type. (XLSX)

S2 Table NGS mapping details corresponding to the supernatants of infected cells. (XLSX)

S3 Table Additional InDel variants outside the canonical GP editing site. (XLSX)
